# Self-regulatory Sex Motives Scale in Online Dating: Exploratory Factor Analysis and Internal Consistency

**DOI:** 10.1177/10778012241243048

**Published:** 2024-04-09

**Authors:** Fatemeh Fereidooni, Judith K. Daniels, Miriam J. J. Lommen

**Affiliations:** Department of Clinical Psychology and Experimental Psychopathology, 3647 University of Groningen, Groningen, the Netherlands

**Keywords:** sex motives for casual sex, risky sex behavior, online dating, childhood maltreatment, sexual victimization

## Abstract

Risky sex behavior is common among online dating users. Understanding the motives behind risky sex behavior might help identify suitable targets for prevention. We developed the Self-regulatory Sex Motives Scale in Online Dating (SSOD) to assess sex motives for casual sex in online dating users. This study evaluated the psychometric properties of the SSOD and examined the relationship between sex motives indexed by the SSOD and risky sex behavior. The new scale showed high internal consistency. Exploratory factor analysis suggested a one-factor solution. Sex motives were related to a higher frequency of having sex on the first date.

The rate of sexual victimization, defined as receiving unwanted sexual acts such as sexual coercion, rape, and other nonconsensual sexual contacts (Reid et al., 2023) is high in community and university student populations. To illustrate, a longitudinal study with a community sample in the United States showed that the rate of victimization over 2 years was 4% for sexual contact and 9.4% for sexual coercion (Testa et al., 2007). The rates for attempted and completed rape were 1.2% and 3.3%, respectively. A cross-sectional study with a large sample recruited from 12 universities in the United States reported a prevalence rate of 24.2% among female students (Jouriles et al., 2022).

One of the factors related to sexual victimization is risky sex behavior, any sexual encounter that increases the risk of sexual victimization (D'Abreu & Krahé, 2016). A study, employing ecological momentary assessment for 42 days, showed that risky sex behavior was a predictor of sexual victimization among college students (Yeater et al., 2022). In a longitudinal study with a 6-month interval, greater expected engagement in risky sex behavior predicted sexual victimization in college students (Combs-Lane & Smith, 2002). Another study with a large sample of community and college student populations found that two risky sex behaviors, that is, exchanging sex for money and lower sexual assertiveness, were related to adulthood sexual victimization (Ullman & Vasquez, 2015).

Cooper et al. (1998) discuss that understanding the motives behind risky sex behavior is important as they might be suitable targets for preventative intervention. To this end, they created a measure, the Motivations for Sexual Intercourse Scale (MSIS), to assess different sex motives including coping with negative emotions and self-affirmation, that is, confirming self-worth. These two motives overlap with self-regulation, defined as an ability to regulate different functions such as emotions and behavior (Raffaelli & Crockett, 2003). Self-regulation is an important factor related to risky sex behavior. For instance, a longitudinal study with a 4-year interval (Raffaelli & Crockett, 2003) reported that low self-regulation in early adolescence predicted sexual risk-taking in late adolescence. Another longitudinal study found that low self-regulation was associated with unprotected sex with nonexclusive dating partners in college students (Quinn & Fromme, 2010). Similarly, several studies provided evidence for subjects using sex as a means of self-regulation, for instance, to cope with negative emotions. This motive showed a positive association with the likelihood of engaging in sex with strangers (Miron & Orcutt, 2014). In addition, using sex for self-affirmation was related to risky sex behavior, sex with strangers, and impulsive sex (Layh et al., 2020). Together, the preliminary evidence points to the importance of self-regulatory sex motives in risky sex behavior. The relevance of these motives becomes even more prominent considering that engagement in risky sex behavior may, in turn, heighten the chance for sexual victimization (Miron & Orcutt, 2014). In line with this, self-regulatory sex motives have not only been found to be related to risky sex but also to adulthood sexual victimization (Fereidooni et al., 2022; Miron & Orcutt, 2014; Myers et al., 2006).

Due to the advance of online dating applications, it might be relevant to study risky sex behavior and its link to sexual victimization in this specific context, too. Online dating is common in adults (31% in a British sample; Cabecinha et al., 2017), but is already relevant in adolescence. In a study with a large sample from different countries, approximately 15% of adolescents and young adults reported the use of online dating (Kaakinen et al., 2021). In terms of sexual risk-taking among online dating users, Choi et al. (2016a) reported that using online dating applications was related to casual and unprotected sex in a community sample. In another study (Beymer et al., 2014), the users of online dating applications were more likely to have two sexually transmitted diseases compared to people who used dating websites or met their sexual partners in person. Regarding sexual victimization, two studies reported that online dating use is associated with a two- to three-fold increase in the risk of sexual victimization among students (Choi et al., 2016; Shapiro et al., 2017).

There is ample evidence that people with a history of childhood maltreatment are at heightened risk for sexual victimization in adulthood, a phenomenon called revictimization. Prior research suggests that childhood sexual abuse is associated with a two- to three-fold increase in the risk of sexual revictimization (Arata, 2002; Jankowski et al., 2002; Van Bruggen et al., 2006). A meta-analysis showed that approximately half of the people with a history of childhood sexual abuse experience sexual revictimization (Walker et al., 2019). Hence, childhood maltreatment might be a risk factor for adulthood sexual victimization in the context of online dating as well. Using sex to cope with negative emotions and to boost self-esteem is a risk factor linking childhood maltreatment to adulthood sexual victimization among online dating users (Fereidooni et al., 2022). This finding is consistent with the results reporting that childhood maltreatment was associated with depression, which in turn was related to adulthood sexual victimization through using sex to regulate negative emotions and expected sex with strangers (Miron & Orcutt, 2014).

Considering the popularity of online dating use, high rates of risky sex behavior and sexual victimization in online dating, the assessment of self-regulatory sex motives among online dating users is important as online dating provides higher chances to encounter potential perpetrators, potentially leading to sexual victimization. Nevertheless, no study, to our knowledge, has examined sex motives underlying risky sex behavior in online dating yet.

The MSIS may be a good candidate for such assessment as it has good psychometric properties (Jardin et al., 2017). However, it needs to be adapted for online dating: several items assess sex motives related to intimacy or connection with partners, while this does not apply to the context of online dating where people have sex with strangers. Furthermore, the items related to self-affirmation are mostly broad (e.g., using sex to feel better or enhance self-confidence) and do not specifically assess how sex helps people to regulate their self-worth.

Based on these limitations, we opted to design a new scale to assess self-regulatory sex motives behind casual sex in online dating. We adopted five items corresponding to “using sex to regulate negative emotions” motive from the MSIS and adjusted them for online dating. We also added six items to measure “using sex for self-affirmation” motive. For the self-affirmation items, we specified how people might feel about themselves by having sex with strangers. This was done by using positive adjectives related to the sense of self, such as “cool,” “brave,” and “adventurous.” Thus, the first aim of the current study was to examine the psychometric properties and factor structure of this new measure, Self-regulatory Sex Motives Scale in Online Dating (SSOD). As a second aim, we tested the hypothesis that higher scores on SSOD are positively correlated with risky sex behavior (i.e., frequency of having sex on the first date with a match).

## Method

The present study was conducted on a database on risk factors of revictimization in online dating. The sample included university students recruited via research platforms and the general population recruited by Qualtrics Company. The study was conducted online via the Qualtrics platform in both the general population and university students. IP addresses were initially recorded to prevent multiple entries of participants but were removed before data analysis*.* First, the participants were informed about the content and potential risks of the study. After informed consent, they responded to the survey in exchange for a monetary reward or research credits depending on the platforms via which they participated. At the end, the participants were debriefed about the aim and hypotheses of the study. The original study was approved by the ethics committee at the University of Groningen.

### Participants

The current study is part of a larger project on mobile dating. The original sample consisted of 413 heterosexual women (*n *= 276 from the community sample and *n *= 137 from university students) aged between 18 and 35 with a mean age of 23.68 (*SD *= 3.62), who reported using mobile dating applications at least one year prior to the study and met at least one match in person. The sample used in the current research consisted of 143 heterosexual women (*n *= 86 from the general population and *n *= 57 from university students) who all reported casual sex with their dating application matches. The inclusion criterion of “casual sex with matches” was critical for the purpose of this study as the aim was to assess sex motives behind casual sex with online dating matches, which can be considered as risky sex behavior. In addition, we limited our sample to heterosexual women since sex motives for casual sex might differ based on sexual orientation and gender. For this first study, we therefore preferred a homogeneous sample of heterosexual women. The mean age of participants was 23.77 (*SD *= 4.26) in the present study. Screening the students based on their childhood maltreatment experiences on the university's research platforms was not allowed. To assure that people with a history of childhood maltreatment are well presented in our sample, we recruited an additional sample in the general population with an additional eligibility criterion; an indication of a positive history of childhood maltreatment assessed by a yes/no question.

### Measures

*Demographic information and information about using mobile dating applications.* The participants reported their age, relationship status, nationality, main motive for using the dating applications, frequency of having sex on the first date with a match, duration of using the dating applications, and the number of matches met in person. Relationship status, nationality, and main motivation for using online dating were asked by multiple-choice questions. The remaining information, including the frequency of having sex on first dates (i.e., frequency of having sex on the first date across the matches), was collected by open-ended questions.

#### Self-Regulatory sex motives scale

Eleven items were used to assess sex motives for casual sex in online dating. We adapted five items from the MSIS and adjusted them for online dating. The remaining items were custom-made (see Table 1). For the custom-made items, we described how one feels when they use sex to regulate their self-affirmation with specific adjectives (e.g., brave, cool, and powerful). In addition, we added an item on engaging in sex to regulate self-affirmation despite being aware that it might be a risky behavior. The participants responded to the SSOD on a visual analog scale (0 = 0% or never, 100 = 100% or in all cases). The means and standard deviations for the items and the whole scale are provided in Table 1.

**Table 1.** Descriptive Statistics for the SSOD Items, Subscales, and Whole Scale.

	*M*	*SD*	Min	Max
1. I have casual sex with matches because I would like to be adventurous.	56.71	27.60	0	100
2. I have casual sex with matches to cope with upset feelings.	33.52	29.24	0	100
3. I have casual sex with matches to help deal with disappointment.	33.58	32.00	0	100
4. I have casual sex with matches because it helps me feel better when I am lonely.	48.31	29.57	0	100
5. I have casual sex with matches because it helps me feel better when feeling low.	45.62	31.22	0	100
6. I have casual sex with matches to cheer myself up.	45.90	30.20	0	100
7. I have casual sex with matches because it makes me feel brave.	40.80	33.40	0	100
8. I have casual sex with matches because it makes me feel powerful.	45.45	32.40	0	100
9. I have casual sex with matches because it makes me feel in control of myself.	46.40	31.00	0	100
10. I have casual sex with matches because it makes me feel cool.	36.75	32.26	0	100
11. In the past, I have decided to engage in a sexual encounter with a match although I was acutely aware that it might be risky because I thought that it would make me feel adventurous or cool or brave.	40.37	31.17	0	100
Total score	473.38	248.26	3.00	1,100

#### Childhood maltreatment

Childhood maltreatment was assessed by the Childhood Trauma Questionnaire-Short Form (CTQ-SF; Bernstein et al., 2003), that measures five forms of childhood maltreatment, that is, physical abuse, sexual abuse, emotional abuse, physical neglect, and emotional neglect. The participants reported these experiences before the age of 15 on a Likert scale (1 = *never true* to 5 = *very often true*). The prevalence of childhood maltreatment was calculated by cutoffs (sexual abuse ≥ 8, physical abuse ≥ 8, physical neglect ≥ 8, emotional neglect ≥ 15, and emotional abuse ≥ 10) recommended by Walker et al. (1999). Previous studies report proper validity and reliability of the CTQ-SF (Bernstein et al., 2003; Gerdner & Allgulander, 2009; Thombs et al., 2009). The internal consistency of the scale in the present sample was excellent *α *= .95.

#### Sexual victimization in online dating

Sexual victimization in the context of online dating was measured by two items for cyber victimization (e.g., “My match sent me unwanted sexual texts although I had clearly told him I did not like that.”) and eight items for in-person sexual victimization (e.g., “My match kissed me although I had clearly told him I did not like that.”). The frequency of sexual victimization for each incident was reported on a visual analog scale (0 = 0% or never, 100 = 100% or in all cases). The internal consistency of this scale was *α *= 90. Endorsement of at least one incident on any item was considered as sexual victimization.

### Data Analysis

First, the assumptions of exploratory factor analysis (EFA) were checked. The relationships between the items were linear and most of the inter-item relationships were sufficiently high (*r *> .30). The normality distribution assumption was not problematic such that the skewness and kurtosis values were in a proper range for all items, skewness ≤ 2.0 and kurtosis ≤ 7.0 as suggested by Watkins (2018) and no outliers were detected. The adequacy of the data for factor analysis was investigated by Bartlett's test of sphericity and Kaiser–Meyer–Olkin measure. The Bartlett's test of sphericity was significant, *X*^2^ (55) = 930.60, *p *< .001. The measure of sampling adequacy (MSA) values for the set of items (*MSA *= .88) and for each item (ranging between .82 and .96) were higher than the cutoff of .50. Therefore, the data was appropriate for EFA. To examine inter-item correlations, we used Pearson correlation and internal consistency was assessed by Cronbach's alpha. The analyses were conducted in the R software environment, Version 4.1.0. The significance threshold of *p *< .05 (two-sided) was used.

To test whether the sex motives are related to the frequency of having sex on the first date, we planned to conduct a Pearson correlation. The assumption check showed that this variable had outliers. Therefore, we decided to run Kendal tau correlation analysis for this variable.

## Results

### Descriptive Statistics

In total, 72.02% (*n *= 103) were single, 20.27% (*n *= 29) in an open relationship, 2.8% (*n *= 4) in a relationship while their partners were not informed that they were using dating applications, and 4.90% (*n *= 7) did not report their relationship status. The majority of participants were from the Netherlands (79.72%, *n *= 114), 11.19% (*n *= 16) were German and the rest (9.09%, *n *= 13) were from various nationalities. The participants’ main motivations for using the applications were finding a serious relationship (51.75%, *n *= 74), casual sex (30.80%, *n *= 44), and meeting new people or making friends (21%, *n *= 15%). The rest (2.8%, *n *= 4) did not report their main motivation. The participants reported that they had met on average 6.07 (*SD *= 7.43) matches in person. The average duration of the dating applications used in months was 11.92 (*SD *= 11.76). The average frequency of having sex on the first date with matches over the course of online dating use was 2.19 (*SD* = 2.62) with the mode being 1.0.

In the whole sample, 72.02% (*n *= 103) reported at least one type of childhood maltreatment. In addition, 74.12% (*n *= 106) reported cyber victimization and 83.22% (*n *= 119) reported in-person sexual victimization. The prevalence of general revictimization, childhood maltreatment accompanied by the indication of either cyber or in-person sexual victimization, was 62.24% (*n *= 89) in the whole sample.

Regarding the subsamples, 86% (*n *= 74) in the general population and 50.9% (*n *= 29) in the student sample indicated at least one type of childhood maltreatment. The rate of cybersexual victimization was reported by 80.2%% (*n *= 69) and 64.9% (*n *= 37) in the general population and students, respectively. This rate was 87.2% (*n *= 75) in the general population and 77.2% (*n *= 44) for in-person victimization. The rate of general revictimization in the community sample and university students were 75.6% (*n *= 65) and 42.1% (*n *= 24), respectively. Table 2 provides information on the prevalence of each form of childhood maltreatment, sexual victimization, and revictimization in the student sample and general population.

**Table 2.** The Prevalence of Childhood Maltreatment, Adulthood Sexual Victimization, and Revictimization in Whole Sample and Subgroups.

		Sample	
	Whole sample *N* (%)	General *N* (%)	Student *N* (%)
**Childhood maltreatment**			
Emotional abuse	90 (62.94)	68 (81)	22(38.6)
Physical abuse	74 (51.75)	65 (79.3)	9 (15.8)
Sexual abuse	67 (46.85)	63 (77.8)	4 (7)
Physical neglect	85 (59.44)	69 (83.1)	16 (29.1)
Emotional neglect	40 (27.97)	33 (40.7)	7 (12.5)
At least one type of childhood maltreatment	103 (72.02)	74 (86)	29 (50.9)
**Sexual victimization**			
Cyber	106 (74.12)	69 (80.2)	37 (64.9)
In-person	119 (83.22)	75 (87.2)	44 (77.2)
General	122 (85.31)	76 (88.4)	46 (80.7)
**Revictimization**			
Childhood and cyber victimization	79 (55.24)	59 (68.6)	20 (35.1)
Childhood and in-person victimization	88 (61.54)	64 (74.4)	24 (42.1)
General	89 (62.24)	65 (75.6)	24 (42.1)

### Inter-item Correlations and Internal Consistency

The correlations between the items of the SSOD are provided in Table 3. The interitem correlations ranged from *r *= .23 to *r *= .74. The majority of interitem correlations were higher than .30. However, Items 1 and 2 showed interitem correlations below .30. We did not exclude these items for further analysis solely based on their interitem correlations. Exclusion of the items did not improve the internal consistency (see Table 3), which also shows good consistency among the items. Therefore, we did not remove any item in spite of the low interitem correlations mentioned above considering that a higher number of items decreases measurement error and potentially enhances criterion validity (Sarstedt & Wilczynski, 2009). The internal consistency for the SSOD was high (*α *= .91, 95% CI [0.89, 0.93]).

**Table 3.** Inter-item Correlation and Internal Consistency if an Item Deleted.

	1	2	3	4	5	6	7	8	9	10	11	*α* if item deleted
1. I have casual sex with matches because I would like to be adventurous.	-	-	-	-	-	-	-	-	-	-	-	.91
2. I have casual sex with matches to cope with upset feelings	.23**	-	-	-	-	-	-	-	-	-	-	.91
3. I have casual sex with matches to help deal with disappointment.	.33**	.74**	-	-	-	-	-	-	-	-	-	.90
4. I have casual sex with matches because it helps me feel better when I am lonely.	.27**	.47**	.48**	-	-	-	-	-	-	-	-	.91
5. I have casual sex with matches because it helps me feel better when feeling low.	.36**	.49**	.61**	.68**	-	-	-	-	-	-	-	.90
6. I have casual sex with matches to cheer myself up.	.35**	.49**	.51**	.57**	.67**	-	-	-	-	-	-	.90
7. I have casual sex with matches because it makes me feel brave.	.49**	.38**	.53**	.42**	.50**	.47**	-	-	-	-	-	.90
8. I have casual sex with matches because it makes me feel powerful.	.45**	.27**	.36**	.44**	.55**	.56**	.69**	-	-	-	-	.90
9. I have casual sex with matches because it makes me feel in control of myself.	.40**	.23**	.37**	.34**	.45**	.37**	.58**	.70**	-	-	-	.91
10. I have casual sex with matches because it makes me feel cool.	.44**	.31**	.44**	.39**	.56**	.53**	.67**	.69**	.54**	-	-	.90
11. In the past, I have decided to engage in a sexual encounter with a match although I was acutely aware that it might be risky because I thought that it would make me feel adventurous or cool or brave.	.42**	.46**	.61**	.49**	.50**	.44**	.61**	.51**	.48**	.62**	-	.90

** *p* < .01.

### Exploratory Factor Analysis

First, parallel analysis was conducted to determine the number of factors to be retained in EFA. Both the scree plot and the eigenvalues (Table 4) suggested a one-factor solution. The results of the factor analysis showed that the factor loadings for all items were high ranging from .54 to .78.

**Table 4.** Eigenvalues in Parallel Analysis and Factor Loadings in EFA.

	Eigenvalues in parallel analysis	Factor loadings in EFA
Item 1	5.38	.54
Item 2	.80	.56
Item 3	.30	.69
Item 4	.02	.64
Item 5	−.01	.76
Item 6	−.03	.71
Item 7	−.09	.78
Item 8	−.12	.78
Item 9	−.24	.66
Item 10	−.24	.78
Item 11	.39	.74

*Note.* EFA = exploratory factor analysis.

### Correlation Analysis

The results showed a small-sized positive relationship between the SSOD scores and the frequency of having sex on the first date (*r *= .16, *p *= .01, 95% CI [0.04, 0.27]).

### Exploratory Analysis

To test the differences between victimized and nonvictimized women in terms of the frequency of having sex on first dates, we conducted Independent-samples *t*-tests. The results showed that women with cyber sexual victimization (*M* = 2.25, *SD* = 2.74) did not differ significantly from women who were not victimized in this context (*M* = 2.03, *SD* = 2.27) with regard to this variable, *t* (141) = .45, *p* = .65. Similar patterns were found for in-person sexual victimization, *t* (141) = 1.17, *p* = .24; frequency in victimized (*M* = 2.31, *SD* = 2.68) versus frequency in nonvictimized (*M* = 1.63, *SD* = 2.42) subjects. However, these null results might be due to the small variance in the frequency of having sex on first dates in the whole sample.

## Discussion

This study examined the psychometric properties of a newly developed scale for assessing self-regulatory motives for having casual sex within the context of online dating. The findings of the study supported the reliability of the SSOD, indexed by a high internal consistency. The results of the factor analysis suggested a one-factor solution for the measure, indicating that the items for emotion and self-affirmation regulatory motives were best represented by a single factor. In line with this finding, an exploratory correlational analysis showed a strong association between the sum scores of the items for regulating negative emotions and the items for enhancing self-affirmation (*r *= .65, *p *< .001). The one-factor solution found in the current study is different from Cooper et al. (1998) findings. In their study, two separate factors in confirmatory factor analysis emerged for items related to coping with negative emotions and regulating self-affirmation. However, the correlation coefficient between the two factors was similar to the one in the present study. The inconsistent findings might be due to differences in the samples in these two studies; the overrepresentation of people with a history of childhood maltreatment in our study. Moreover, the motives for casual sex in online dating might differ from motives in other contexts.

We also found that higher levels of self-regulatory sex motives were associated with a higher frequency of having sex on the first date across the matches. This finding is in line with Miron and Orcutt’s (2014) results that showed a relationship between emotion regulation sex motive and higher likelihood of sex with strangers in a path analysis model. The association in the present study was weak. The frequency of having sex on the first date was low (between 0 and 1) in the majority of the sample (*n *= 61, 80.70%); the limited variance in this variable might also explain the relatively weak association between the frequency of having sex on the first date and the presence of sex motives. Both sex motives and risky sex behavior were examined cross-sectionally in this study. Therefore, it is important to understand how the sex motives in the SSOD would predict risky sex behavior in future prospective studies especially since sexual risk-taking is a risk factor for revictimization (Krahé & Berger, 2017; Testa et al., 2010).

The results of the study should be interpreted with caution as we oversampled people with childhood maltreatment. This might have influenced the factor structure of the SSOD in the current sample. In addition, a high prevalence of childhood maltreatment could result in less variation in sex motives and risky sex behavior which in turn might contribute to an underestimation of the relationship between sex motives and risky sex behavior (as reflected in a high frequency of having sex on the first date).

### Limitations of the Study

The current study has some important limitations that need to be considered when interpreting the current findings. The psychometric assessment of the SSOD was limited to evaluating its factor structure and its internal consistency. To further our knowledge about the validity and reliability of the SSOD, research is needed on the convergent validity and test–retest reliability of the scale. Although the sample size of the study was adequate for factor analysis (de Winter et al., 2009), the sample was a combined sample consisting of individuals from the general population and university students. The factor structure and factor loadings might be different for these two populations. Invariance measurement can help us understand potential differences between these populations. However, due to the limited sample size in each group, measurement invariance could not be reliably assessed in the current study. Moreover, it should be acknowledged that implementing the inclusion criterion of “positive history of childhood maltreatment” in the community sample, but not in the student sample might further hamper a meaningful comparison of both samples in our study. Future studies with similar inclusion criteria for both samples can resolve the issue. In addition, the sample of this study was limited to heterosexual women in their young adulthood and the results might not be generalizable to other populations. Examining the factor structure and psychometric properties of the SSOD in other populations can provide information about the relevance of this measure in different age, gender, and sexual orientation groups. Given the previously reported differences between men and women regarding their sex motives (Cooper et al., 1998), the factor structure of SSOD needs to be examined in other genders as well to understand whether the emerged factor structure in women generalizes to men. In addition, given the higher risk of sexual victimization among homosexual populations (Hughes et al., 2010; Kuyper & Vanwesenbeeck, 2011), higher number of sexual partners, and the effects of experiencing minority stress (i.e., hiding one's sexual orientation and having negative attitudes toward one's own sexual orientation) on sexual victimization in such populations (Kuyper & Vanwesenbeeck, 2011), gaining insights into sex motives in the context of online dating in these populations is pivotal and we call on future studies to address this.

### Strengths of the Study

The current study has several strengths. To our knowledge, this is the first study examining the psychometric properties of a scale assessing motives for casual sex among online dating users. These motives might help us to understand why people engage in risky sex behavior in the context of online dating. Another strength of the study was assessing sex motives in a sample in which the majority of participants reported high severity of childhood maltreatment. Assessing the psychometric properties and factor structure of the SSOD in such a sample is informative because risky sex behavior is prevalent among people with a history of childhood maltreatment (Wilson & Widom, 2008). Furthermore, the inclusion of the items explaining how people might specifically feel about themselves using casual sex might give more precise information about self-regulatory sex motives, as the items provide specific positive adjectives for self-worth. This adjustment of the scale is an improvement compared to the original MSIS, in which the items provided a general and broad explanation of increasing self-esteem using sex. In addition, the new scale is shorter compared to the MSIS and, importantly, includes sex motives that are relevant to the context of online dating.

### General Conclusion

In sum, the findings of the study provide support for the internal consistency of the SSOD and suggest a one-factor solution for this measure. In addition, the findings provided preliminary support for the hypothesis that self-regulatory sex motives are associated with risky sex behaviors among online dating users. Together, the findings support the relevance of this brief 11-item scale as a measure of self-regulatory motives for having casual sex among online dating users. Future studies can further our knowledge about the validity and test–retest reliability of the scale as well as its factor structure in populations other than heterosexual women in their young adulthood.
